# Shenmai Injection Improves Energy Metabolism in Patients With Heart Failure: A Randomized Controlled Trial

**DOI:** 10.3389/fphar.2020.00459

**Published:** 2020-04-17

**Authors:** Shao-mei Wang, Li-fang Ye, Li-hong Wang

**Affiliations:** ^1^ Cardiovascular Medicine Department, People’s Hospital of Hangzhou Medical College, Hangzhou, China; ^2^ Bengbu Medical College, Bengbu, China

**Keywords:** heart failure, energy metabolism, Shenmai injection, cardiac function, traditional Chinese medicine

## Abstract

**Background:**

In recent years, the application of Shenmai (SM) injection, a traditional Chinese medicine (TCM), to treat heart failure (HF) has been gradually accepted in China. However, whether SM improves energy metabolism in patients with HF has not been determined due to the lack of high-quality studies. We aimed to investigate the influence of SM on energy metabolism in patients with HF.

**Methods:**

This single-blind, controlled study randomly assigned 120 eligible patients equally into three groups receiving SM, trimetazidine (TMZ), or control in addition to standard medical treatment for HF for 7 days. The primary endpoints were changes in free fatty acids (FFAs), glucose, lactic acid (LA), pyroracemic acid (pyruvate, PA) and branched chain amino acids (BCAAs) in serum. The secondary outcomes included the New York Heart Association (NYHA) functional classification, TCM syndrome score (TCM-s), left ventricular injection fraction (LVEF), left ventricular internal diastolic diameter (LVIDd), left ventricular internal dimension systole (LVIDs), and B-type natriuretic peptide (BNP).

**Results:**

After treatment for 1 week, the NYHA functional classification, TCM-s, and BNP level gradually decreased in the patients in all three groups, but these metrics were significantly increased in the patients in the SM group compared with those in the patients in the TMZ and control groups (P < 0.05). Moreover, energy metabolism was improved in the NYHA III–IV patients in the SM group compared with those in the patients in the TMZ and control groups as evidenced by changes in the serum levels of FFA, LA, PA, and BCAA.

**Conclusions:**

Integrative treatment with SM in addition to standard medical treatment for HF was associated with improved cardiac function compared to standard medical treatment alone. The benefit of SM in HF may be related to an improvement in energy metabolism, which seems to be more remarkable than that following treatment with TMZ.

## Introduction

Heart failure (HF) is a clinical syndrome characterized by impaired cardiac function due to various cardiovascular diseases and has a mortality of approximately 50% (Tu et al., 2009). Although progress has been achieved in HF treatment in recent years, the 5-year mortality of patients with HF remains high (Benjamin et al., 2017). Current evidence-based treatments for HF mainly include neurohormonal inhibitors, such as medications targeting the renin-angiotensin aldosterone system and the β-adrenergic receptor signaling pathway, mineral corticosteroid receptor antagonists, medications that improve the HF syndrome, such as inositol and diuretics, and mechanical devices (Ponikowski et al., 2016). A recent understanding of the pathophysiological mechanisms of HF highlighted the potential importance of biogenesis dysfunction (Neubauer, 2007). Increasing evidence indicates that a disruption in myocardial energy metabolism in patients with HF leads to the progression and deterioration of the disease (Stanley et al., 2005; Bertero and Maack, 2018). Previous studies suggested the concept of metabolic remodeling in the failing myocardium (van Bilsen et al., 2004) in which the metabolic pathways that regulate energy in the heart are changed in the event of myocardial failure, and the main reason for this change is the disordered metabolism of myocardial lipids, glucose, and other substances, ultimately leading to impaired cardiac function and structure (Evans and Clarke, 2012; Lopaschuk and Ussher, 2016). Accordingly, improvement in cardiac energy metabolism has been proposed as a potential treatment for HF. Recently, treatment with some drugs that directly improve myocardial energy metabolism has been shown to benefit cardiac function in HF patients (Birkenfeld et al., 2019). Trimetazidine (TMZ), which functions by shifting the substrates of cardiac energy metabolism from free fatty acid (FFA) to glucose (Fragasso et al., 2006), has been shown to improve cardiac function and reduce whole body resting energy expenditure (REE) in HF (Fragasso et al., 2011; Lopatin, 2015). Additionally, the use of TMZ in Chronic heart failure (CHF) patients may decrease hospitalization for cardiac causes, improve clinical symptoms and cardiac function, and simultaneously ameliorate left ventricular remodeling (Zhang et al., 2012). Given the unsatisfactory clinical efficacy of the current treatments for HF, novel medications targeting the disordered energy metabolism in the heart remain to be developed.

From the perspective of traditional Chinese medicine (TCM), HF is mainly caused by Qi deficiency and either Yin or Yang inadequacy (Mao et al., 2009; Chen et al., 2012; Tang and Huang, 2013). In this era, Shenmai (SM) injection, “a TCM consisting of Panax ginseng C.A.Mey. and Ophiopogon japonicus (Thunb.) Ker Gawl.” and, has been used to treat patient with qi-yin deficiency for over 1,500 years in China. After receiving approval by the China Food and Drug Administration (CFDA), SM is an effective and safe agent used to treat CHF (Zhang et al., 2002; Long et al., 2003). Some previous studies have shown that SM has been used to treat patients with chronic cor-pulmonale (Ouyang et al., 2006; Lu and Zheng, 2014; Shi et al., 2015), and acute and chronic HF (Chen et al., 2012) with obvious clinical effects. Regarding HF, SM has been shown to improve the left ventricular injection fraction (LVEF) (Ma et al., 2010; Chen et al., 2014). The benefits of SM on cardiac function in HF and chronic cor-pulmonale have also been indicated in a previous systematic review (Li et al., 2011). However, the pharmacological mechanisms underlying the benefits of SM in HF remain unclear. It has been suggested based on animal studies that SM and its components can restore energy metabolism and mitochondrial function in HF models (Wang et al., 2018). However, the influences of SM on cardiac energy metabolism in HF patients remain unclear. The aim of the current study was to compare the influences of SM, TMZ, and blank controls on cardiac function and markers of energy biogenesis in HF patients on the basis of standard medical treatment for HF.

## Material and Methods

### Study Design

This study was designed as a randomized, double-blind, controlled study. The trial was conducted in Zhejiang Provincial People’s Hospital and enrolled 120 patients with HF based on the inclusion and exclusion criteria. The study was performed in accordance with the Declaration of Helsinki and good clinical practice and national regulations. The protocol was approved by the local ethics committee before performing the study, and informed consent was obtained from all participants. The protocol of this study was registered at the Chinese Clinical Trial Registry (ChiCTR1800016293).

Eligible patients were randomly assigned to the SM group, TMZ group, or control group at a ratio of 1:1:1. The randomization was performed with a computer-generated random list by SAS software (SAS Institute, Cary, North Carolina). All patients and detectors were blind to the allocation.

All inpatients with HF that met the inclusion and exclusion criteria received standard medicines according to the Chinese Society of Cardiology guidelines for the treatment and diagnosis of HF. In addition to standard medicines, the eligible patients were randomly assigned to groups that received intravenous SM injection (100 ml/day) at the rate of 20–40 drops per min, oral TMZ treatment two times per day (70 mg/day total, 35 mg each dose) or standard treatment without SM and TMZ (control) for 7 consecutive days. At baseline, before the intervention, the patients were evaluated. After treatment for 7 days, the effects of the intervention on the patients were evaluated. Except for SM or TMZ, any other medicine that could confound the outcome of the SM and TMZ treatments was forbidden. In the case of comorbidities, only medicines that fulfilled the corresponding guidelines were allowed.

### Study Materials

SM injection is a TCM extracted from Panax ginseng (Panax ginseng C.A. Mey, steamed and dry) and Ophiopogon japonicus

[Ophiopogon japonicus (L.f.) Ker-Gawl, root]. SM injection is mass produced as a patented drug based on the national standards approved by the CFDA from Chia Tai Qingchunbao Pharmaceutical Co., Ltd. (Hangzhou, China). TMZ was purchased from Servier Industry Laboratories (France). The Assay kits used to measure the glucose, FFA, LA, and PA contents were purchased from Solarbio Science & Technology Co., Ltd. (Beijing, China). The BCAA kit was obtained from Sigma-Aldrich Co., LLC (USA). Acetonitrile of high-performance liquid chromatography (HPLC) grade was obtained from Fisher Scientific (New Jersey, USA), and purified water was purchased from Robust Company (Hangzhou, China).

### Quantitative Analysis of SM

The main SM components were quantitated by a HPLC fingerprint analysis in combination with pattern recognition techniques on an Agilent 1290 instrument (Agilent, America) as previously described (Lu et al., 2012) according to the Chinese Pharmacopoeia (2010) with slight modifications. All components were separated on a Diamond C18 column (4.6 mm × 250 mm, 5 μm) at a flow rate of 1.0 ml/min. The column temperature was 30°C, and the wavelength was set to 203 nm. The mobile phase comprised 0.1% phosphate in pure water (A) and acetonitrile (B), and a gradient elution was performed as follows: 10 min (90%, A), 25 min (80%, A), 60 min (60%, A). All data were processed using Open LAB CDS Chemstation (Agilent, America). The batch sample analyses of SM and related characteristic records were maintained as previously reported (Wu et al., 2013).

### Study Participants

In accordance with the 2014 Chinese guidelines for the diagnosis and treatment of HF, the inclusion criteria for the study were as follows: male and female patients with HF; New York Heart Association (NYHA) functional classification of II–IV; unstable condition requiring further treatment in the hospital; and the submission of an informed consent form. The exclusion criteria were as follows: severe cognitive impairment or other causes of inability to communicate; comorbidities of acute myocardial infarction, cardiac shock, or severe arrhythmia with hemodynamic changes; other chronic diseases with a serious impact on the quality of life; therioma; chronic obstructive pulmonary disease; severe liver dysfunction (index value of liver function > 2 times the normal value); renal insufficiency (creatinine clearance rate > 20%, serum creatinine > 3 mg/dl or >265 μmol/L); cerebral apoplexy; pregnancy or breastfeeding; participation in other clinical trials within 1 month; and refusal to use or allergic to SM or TMZ.

### Clinical Endpoints

The primary endpoints were changes in FFAs, glucose, lactic acid (LA), pyroracemic acid (pyruvate, PA) and branched chain amino acids (BCAAs) in serum. The secondary outcomes included the NYHA functional classification, TCM syndrome score (TCM-s), LVEF, left ventricular internal diastolic diameter (LVIDd), left ventricular internal dimension systole (LVIDs) and B-type natriuretic peptide (BNP). The NYHA classification and TCM-s were evaluated by two researchers who were not involved in the treatment of the subgroup of patients. The echocardiographers were blinded to the study intervention.

### Laboratory Tests

Routine blood tests, including creatinine, alanine aminotransferase (ALT) and aspartate aminotransferase (AST), were performed for each patient. The levels of glucose, FFAs, LA, PA, and BCAAs in the serum were detected according to the instructions of the related assay kits.

### Sample Size

According to the primary outcome of NYHA function, with a significance level of 0.05, estimated statistical power of 0.80, and differences in NYHA function over 1 week of 15%, the estimated sample size was 32 per group. After considering the requirement of CFDA for drug registration and an approximate dropout rate of 20%, in total, 120 patients (40 per treatment group) were needed for this study.

### Statistical Analysis

For the normally distributed continuous variables, the means ± SD are used to summarize the data; for BNP, which is not normally distributed, a logarithm base 10-transform was performed to normalize the within group distributions. We used paired t-tests to compare the continuous variables, and chi-square tests to analyze the discrete variables. Variables without a Gaussian distribution (as assessed by the Kolmogorov-Smirnov test) were compared by the Wilcoxon test (NYHA class and medications). Kruskal-Wallis one-way analysis of variance (ANOVA) of ranks and ANOVA for repeated measures with one factor (baseline-7 days) were used to assess the treatment effect on the variables in the different groups (BNP, TCM-s, ALT, AST, creatinine, glucose, FFA, LA, PA, and BCAA). All calculated P-values were two-tailed and considered significant at <0.05. We used SPSS 19 for the statistical analyses.

## Results

### Quantitation of SM

**Figure 1:**
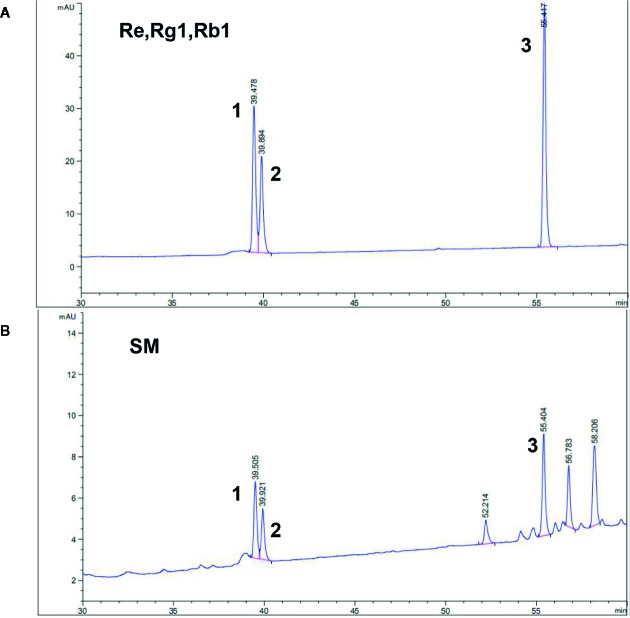
HPLC fingerprint of Shenmai injection and the ginsenosides Re, Rg1, and Rb1 as reference standards. A. Chromatogram of the reference standards: 1 represents ginsenoside Re; 2 represents ginsenoside Rg1; 3 represents ginsenoside Rb1. B. Representative chromatogram of Shenmai injection samples: No. 1–3 represent the common peaks with the reference standards Re, Rg1, and Rb1.

According to the reference standards’ fingerprint, the peak areas of the ginsenosides Re, Rg1, and Rb1 at the corresponding retention times (39.478, 39.894, and 55.417 min, respectively) were used to calculate the total contents of Re, Rg1, and Rb1 in SM as 0.3 mg/ml. The ginsenosides Re, Rg1, and Rb1 were the major bioactive components of TSPG. The HPLC chromatogram of SM including ginsenosides as a standard is shown in **Figure 1**, and the SM fingerprint shows that the TSPG content in SM represented by the total contents of Re, Rg1, and Rb1 was 0.3 mg/ml. The HPLC chromatogram of SM standardized as ginsenosides is presented in **Figure 1**. The production of the components was consistent with the national standards for SM injection approved by the CFDA.

### Patient Characteristics

**Figure 2:**
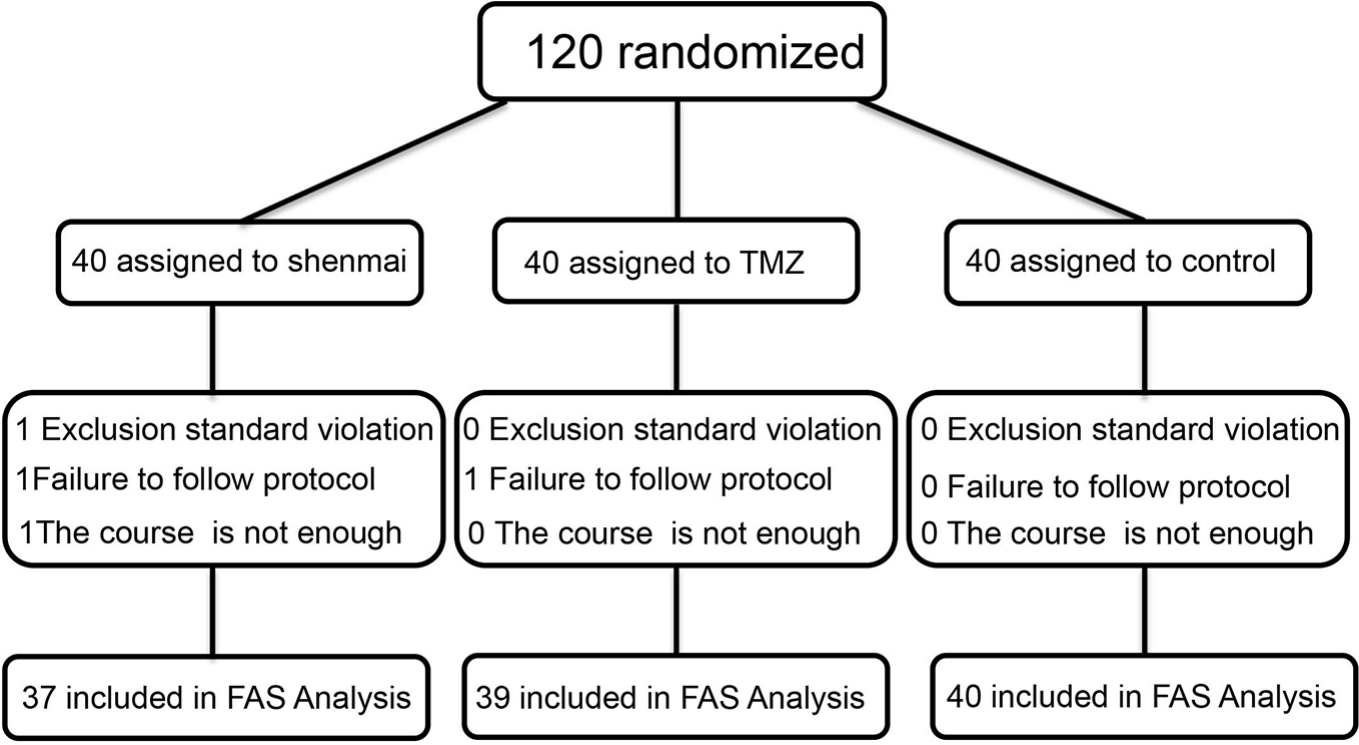
Flow chart of the patients included throughout the study. FAS, full analysis set.

The patients were enrolled between July 2018 and May 2019 as summarized in **Figure 2**. Among the 120 included patients, 116 patients (SM, n=37; TMZ, n=39; control, n=40) were eligible for the primary analysis. One patient (SM, n=1; TMZ, n=0; control, n=0) was excluded from the primary analysis due to a violation of an exclusion standard. Another two patients (SM, n=1; TMZ, n=1; control, n=0) were excluded because of protocol deviations that could affect the efficacy assessments. The most common reason for exclusion was discontinuation of SM or TMZ due to a change in the disease. One patient (SM, n=1; TMZ, n=0; control, n=0) was excluded from treatment due to discharge or transfer. None of the study subjects discontinued SM and TMZ due to side effects, and the dose was not modified during the study. The demographic and baseline characteristics of the patients in the SM, TMZ, and control groups were well balanced (**Tables 1** **and** **2**).

**Table 1:** Baseline characteristics of the patients enrolled in the study.

Characteristics	SM (n=37)	TMZ (n=39)	Control (n=40)	P-value
Sex (men)	23(62.2%)	24(61.5%)	24(60.0%)	0.980
Age	71.27 ± 11.25	69.74 ± 14.06	71.93 ± 12.72	0.705
Heart rate (beats/min)	87.89 ± 20.73	84.62 ± 18.63	82.30 ± 18.56	0.447
HBP	24(64.9%)	19(48.7%)	20(50.0%)	0.297
Diabetic	7(18.9%)	13(33.3%)	10(25.0%)	0.356
NYHA class				
I	0(0.0%)	0(0.0%)	0(0.0%)	0.448
II	4(10.8%)	4(10.3%)	2(5.0%)	
III	22(59.5%)	29(74.4%)	29(72.5%)	
IV	11(29.7%)	6(15.4%)	9(22.5%)	
≥III	33(89.2%)	35(89.7%)	38(95%)	0.300
LVIDd	57.81 ± 11.07	62.33 ± 9.81	60.75 ± 12.14	0.201
LVIDs	45.38 ± 11.97	51.33 ± 10.64	47.75 ± 14.54	0.117
LVEF (%)	43.05 ± 12.34	40.33 ± 9.48	40.63 ± 12.47	0.531
LVEF (≤50%)	30(81.1%)	35(89.7%)	31(77.5%)	0.339
LVEF (% of patients with LVEF ≤ 50%)	38.53 ± 8.19	38.31 ± 7.69	35.48 ± 8.32	0.265
TCM-s	36.38 ± 9.69	37.72 ± 9.29	36.18 ± 10.13	0.771
**Medications**				
ACEI-ARB-NVR	26(70.3%)	33(84.6%)	30(75.0%)	0.321
RH-BNP	4(10.8%)	6(15.4%)	7(17.5%)	0.702
Milrinone	19(51.4%)	23(59.0%)	22(55.0%)	0.801
Beta-blocker	29(78.4%)	34(87.2%)	34(85.0%)	0.563
Digoxin	14(37.8%)	17(43.6%)	17(42.5%)	0.866
Loop diuretic	36(97.3%)	39(100.0%)	40(100.0%)	0.344
Calcium channel blockers	4(10.8%)	4(10.3%)	8(20.0%)	0.374
ADP receptor blocker	20(54.1%)	19(48.7%)	21(52.5%)	0.892
Antibiotic	26(70.3%)	26(66.7%)	27(67.5%)	0.941
Statins	25(67.6%)	24(61.5%)	24(60.0%)	0.773

Values are expressed as the mean ± SD or n (%). HBP, High Blood Pressure; NYHA, New York Heart Association; LVIDd, Left Ventricular Internal Diastolic Diameter; LVIDs, Left Ventricular Internal Dimension Systole; LVEF, Left Ventricular Ejection Fraction; TCM-s, Traditional Chinese Medicine Syndrome Score; ACEI, Angiotensin Converting Enzyme Inhibitor; ARB, Angiotensin-II Receptor Blockers; ADP, Adenosine Diphosphate; NVR (SVST), Sacubitril Valsartan Sodium Tablets.

**Table 2:** Laboratory measurements of the patients enrolled in the study.

Laboratory measurements		SM (n=37)	TMZ (n=39)	Control (n=40)	P-value
ALT (U/l)	baseline	28.24 ± 24.84	25.23 ± 14.45	25.20 ± 21.99	0.547
	7 d	29.00 ± 21.77	22.15 ± 11.15	22.88 ± 17.09	0.406
AST(U/l)	baseline	31.35 ± 21.03	32.85 ± 15.59	32.28 ± 22.11	0.197
	7 d	32.65 ± 20.25	29.18 ± 11.86	28.80 ± 14.80	0.552
Creatinine (μmol/l)	baseline	110.40 ± 40.24	104.97 ± 40.73	112.20 ± 33.92	0.381
	7 d	104.58 ± 34.71	106.72 ± 44.51	112.04 ± 41.97	0.614
FFA (μmol/l)	baseline	670.73 ± 228.75	645.39 ± 222.14	689.58 ± 235.65	0.692
	7 d	469.60 ± 231.33	526.77 ± 212.51	562.73 ± 207.60	0.172
Glucose (mmol/l)	baseline	5.40 ± 1.26	5.81 ± 2.31	5.38 ± 1.25	0.848
	7 d	5.13 ± 0.90	5.71 ± 1.80	5.43 ± 1.73	0.446
LA (mmol/l)	baseline	2.48 ± 0.98	2.22 ± 0.75	2.13 ± 0.75	0.168
	7 d	1.95 ± 0.67	1.89 ± 0.62	1.75 ± 0.52	0.308
PA (μg/ml)	baseline	17.09 ± 8.22	16.56 ± 6.23	16.61 ± 10.19	0.595
	7 d	14.55 ± 7.47	15.47 ± 6.39	15.84 ± 9.45	0.461
BCAA (nmol/μl)	baseline	0.46 ± 0.17	0.44 ± 0.17	0.45 ± 0.21	0.925
	7 d	0.55 ± 0.16	0.46 ± 0.14	0.48 ± 0.17	0.053
BNP (log10)^a^	baseline	2.87 ± 0.36	2.97 ± 0.38	2.92 ± 0.42	0.538
	7 d	2.32 ± 0.40	2.48 ± 0.47	2.57 ± 0.48	0.060

Values are expressed as the mean ± SD. ALT, Alanine Transaminase; AST, Aspartate Transaminase; FFA, Free Fatty Acids; LA, Lactic Acid; PA, Pyroracemic Acid; BCAA, Branched Chain Amino Acid; BNP, B-type Natriuretic Peptide. ^a^BNP level was logarithm base 10-transformed before the analysis.

### NYHA Functional Classification

**Figure 3:**
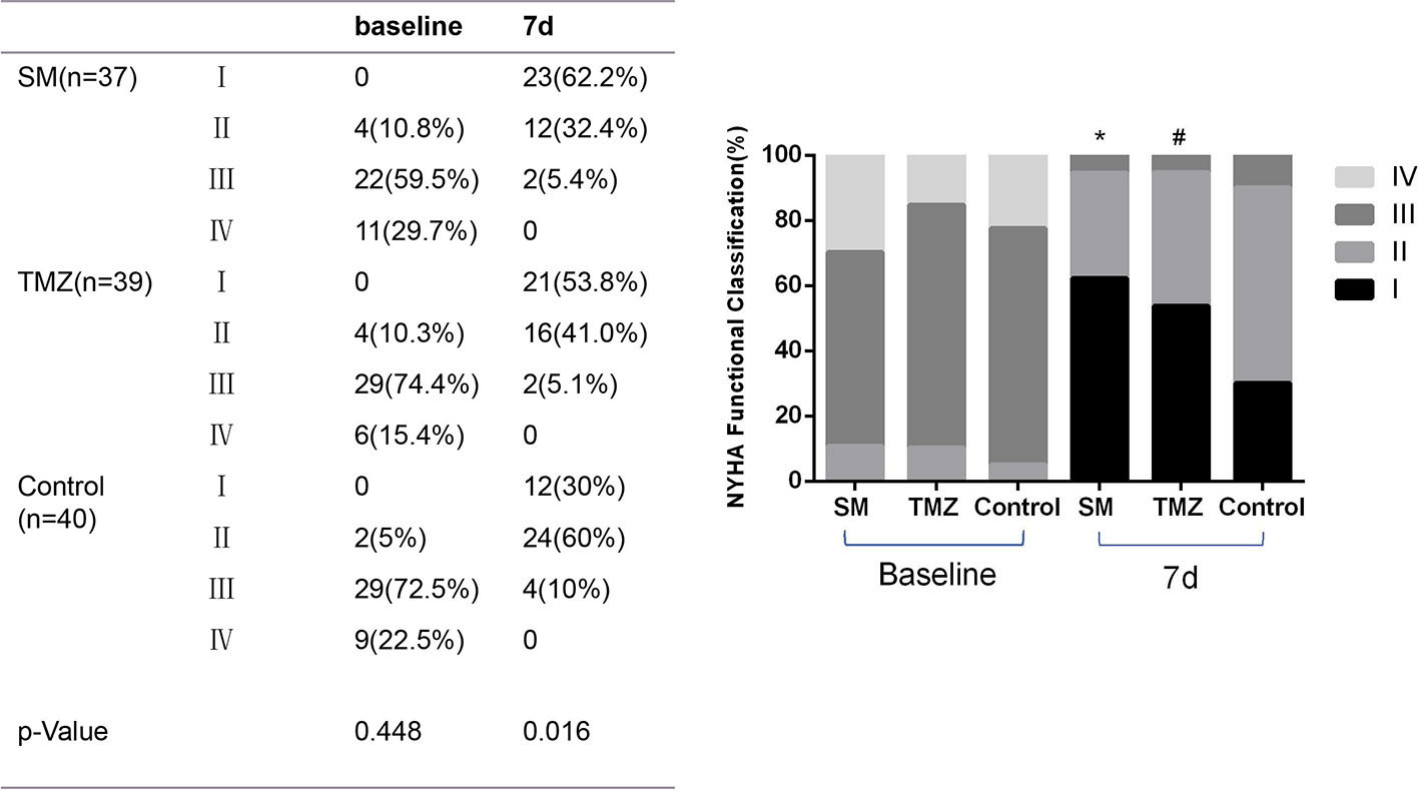
The effects of the SM, TMZ, and control treatments on the NYHA functional classification in HF patients. *P < 0.01 and ^#^P < 0.05 vs. the control (7 days), *P < 0.01 compared with the TMZ group (7 days).

Most enrolled patients were classified as NYHA III to IV (91.4%), and the remaining patients were classified as NYHA II (8.6%). The functional classification of the three groups at baseline was similar (**Table 1**). After the treatment with SM, TMZ, or control plus standard drugs, the frequency of patients with NYHA I and II gradually increased, while the frequency of the patients with NYHA III and IV continuously decreased (**Figure 3**). After 7 days, the proportion of NYHA I and NYHA II patients increased by 62.2 and 32.4% in the SM group, 53.8 and 41.0% in the TMZ group, and 30 and 60% in the control group, respectively. In addition, the proportion of NYHA III–IV patients decreased by 54.1 and 29.7% in the SM group, 69.3 and 15.4% in the TMZ group, and 62.5 and 22.5% in the control group, respectively. Compared to the control treatment, the NYHA function classification was more significantly improved following the treatment with SM and TMZ. Furthermore, the effect of SM was more remarkable than that of TMZ (P < 0.01).

### Changes in BNP

**Figure 4:**
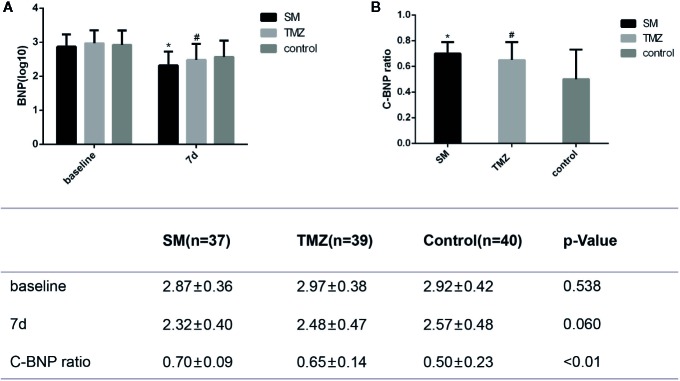
The effects of the SM, TMZ, and control treatments on C-BNP in HF patients. **(A)** Levels of BNP at baseline and after 7 days of treatment in the SM, TMZ, and control groups; *P <0.01 vs. the baseline of SM; ^#^P < 0.01 vs. the baseline of TMZ; the control group (7 days), P < 0.01 vs. the baseline of control; *P < 0.05 and ^#^P > 0.05 compared with the control group (7 days); **(B)** C-BNP ratio in the SM, TMZ, and control groups after 7 days; *P < 0.01 or ^#^P < 0.01 vs. the control group (7 days). C-BNP ratio=log_10_(10^baseline^-10^7d^)/baseline. 7d, 7days.

As shown in **Figure 4**, the patients in the three groups had similar levels of BNP at baseline (P=0.538). After treatment for 7 days, BNP was significantly decreased in all groups (SM, P < 0.01; TMZ, P < 0.01; control, P < 0.01). On day 7, the BNP level in the SM group and TMZ group was decreased from baseline to 2.32 ± 0.40 and 2.48 ± 0.47, respectively, whereas the BNP level in the control group was 2.57 ± 0.48. Compared with the control treatment, the SM treatment significantly reduced the levels of BNP (P < 0.05, **Figure 4A**). However, the effects of TMZ and the control on BNP did not significantly differ. In addition, the changes in the BNP (C-BNP) ratio in the SM and TMZ groups were significantly larger than those in the control group (P < 0.01, **Figure 4B**).

### Changes in TCM-s

**Figure 5:**
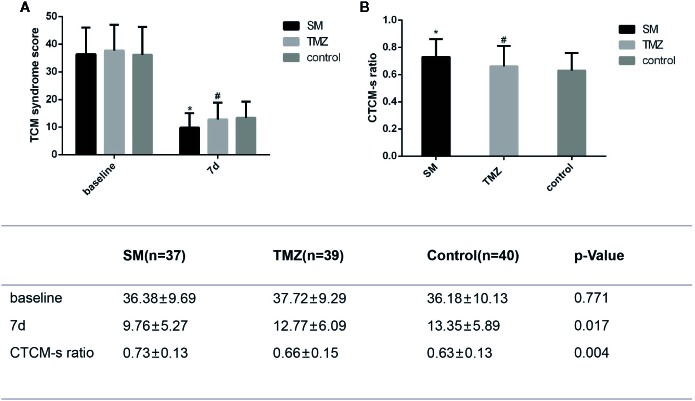
The effects of the SM, TMZ, and control treatments on CTCM-s in patients with HF. **(A)** CTCM-s at baseline and after 7 days of treatment in the SM, TMZ, and control groups; *P <0.01 vs. the baseline of SM; ^#^P < 0.01 vs. the baseline of TMZ; the control group (7 days), P < 0.01 vs. the baseline of control; *P < 0.01 and ^#^P > 0.05 compared with the control group (7 days), *P < 0.05 compared with the TMZ group (7 days); **(B)** CTCM-s in the SM, TMZ, and control groups after 7 days; *P < 0.01 or ^#^P > 0.05 vs. the control group (7 days); *P < 0.05 vs. the TMZ group (7 days). CTCM-s ratio= (baseline-7 days)/baseline.

After treatment for 7 days, the TCM-s was significantly decreased in the SM, TMZ, and control groups (**Figure 5A**). Furthermore, SM improved the TCM-s more remarkably than the control treatment (P < 0.01) and TMZ treatment (P < 0.05). The changes in the traditional Chinese medicine syndrome score (CTCM-s) from baseline over 7 days in the SM group were more remarkable than those in the TMZ and control groups (**Figure 5B**). Moreover, the effects of the TMZ treatment did not significantly differ from those of the control treatment (P > 0.05).

**Figure 6:**
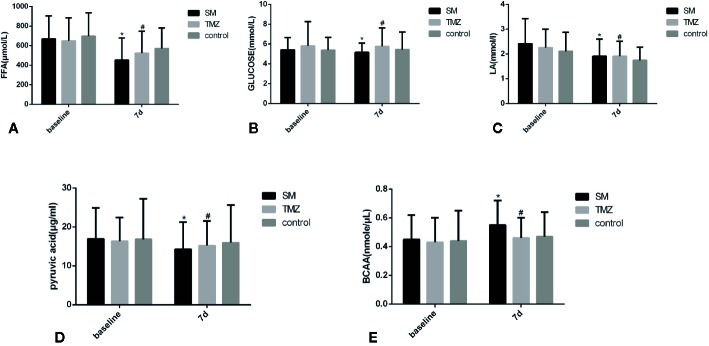
The effects of the SM, TMZ and control treatments on metabolism substrates in the serum in patients with HF. **(A)** Changes in serum FFAs in patients with HF; *P <0.01 vs. the baseline of SM; ^#^P < 0.05 vs. the baseline of TMZ; control group (7 days), P < 0.05 vs. the baseline of control; *P < 0.05 and ^#^P > 0.05 compared with the control group (7 days). **(B)** Changes in serum glucose in patients with HF; *P > 0.05 or ^#^P > 0.05 compared with the control group (7 days). **(C)** Changes in serum LA in patients with HF; *P <0.05 vs. the baseline of SM; ^#^P < 0.05 vs. the baseline of TMZ; the control group (7 days), P < 0.05 vs. the baseline of control; *P > 0.05 or ^#^P > 0.05 compared with the control group (7 days). **(D)** Changes in serum PA in patients with HF; *P <0.05 vs. the baseline of SM; *P > 0.05 or ^#^P > 0.05 compared with the control group (7 days). **(E)** Changes in serum BCAAs in patients with HF; *P <0.05 vs. the baseline of SM; *P < 0.05 or ^#^P > 0.05 compared with the control group (7 days).

Among the enrolled patients with NYHA Class ≥III, the serum metabolic energy substrates FFA, glucose, LA, PA and BCAA did not differ among the groups at baseline (**Table 3**). After treatment for 7 days, the contents of FFA and LA in the serum in each group were significantly lower than those at baseline (**Figures 6A, C**), but the levels of glucose did not change (**Figure 6B**). In the SM group, the PA levels significantly decreased after 7 days compared with baseline (P < 0.05), but no difference was detected in the TMZ and control groups (**Figure 6D**). A trend towards lower levels of FFA and LA was observed on day 7 in each group. The FFA level in the SM group was significantly lower than that in the control group (P < 0.05), but there was no difference in the serum contents of FFA and LA between the TMZ group and the control group (**Figures 6A, C**). Additionally, the BCAA content in the SM group was significantly increased after treatment for 7 days, and the BCAA levels in the SM group were significantly higher than those in the TMZ and control groups; however, there were no significant changes in the BCAA content in the TMZ and control groups after 7 days of treatment (**Figure 6E**).

**Table 3:** Energy metabolism substrates in the serum of patients enrolled with NYHA ≥ III.

Laboratory Measurements		SM (n=33)	TMZ (n=35)	Control (n=38)	P-value
FFA (μmol/l)	baseline	668.49 ± 235.13	649.80 ± 234.30	697.42 ± 239.20	0.687
	7 d	452.88 ± 226.62	523.09 ± 224.06	571.42 ± 209.40	0.081
Glucose (mmol/l)	baseline	5.41 ± 1.23	5.81 ± 2.44	5.38 ± 1.28	0.975
	7 d	5.15 ± 0.95	5.76 ± 1.87	5.44 ± 1.77	0.415
LA (mmol/l)	baseline	2.41 ± 1.01	2.25 ± 0.75	2.11 ± 0.76	0.315
	7 d	1.91 ± 0.69	1.91 ± 0.60	1.74 ± 0.53	0.387
PA (μg/ml)	baseline	16.93 ± 7.96	16.34 ± 6.10	16.82 ± 10.41	0.697
	7 d	14.29 ± 6.93	15.20 ± 6.34	15.94 ± 9.69	0.552
BCAA (nmol/μl)	baseline	0.45 ± 0.17	0.43 ± 0.17	0.44 ± 0.21	0.892
	7 d	0.55 ± 0.17	0.46 ± 0.14	0.47 ± 0.17	0.065

Values are expressed as the mean ± SD.

## Discussion

To the best of our knowledge, the present study represents the most comprehensive evaluation of the efficacy of SM on systematic energy metabolism in patients with HF. The efficacy and safety of SM in chronic HF have been reported in many previous studies (Chen et al., 2014; Zhou et al., 2014). Previous studies have shown that SM combined with concurrent HF medication is better than concurrent HF medication alone for the improvement of 6-min walk test results, ejection fraction, BNP level, etc. Our previous study showed that SM and TMZ improve energy metabolism in primary cardiomyocytes after ischemia-reperfusion (Wang et al., 2019a), highlighting the beneficial effects of SM on energy metabolism in HF. In HF patients, the improvement in functional class and LV function induced by the TMZ treatment is associated with a reduction in whole body REE due to the inhibition of fatty acid oxidation (Fragasso et al., 2011; Lopatin et al., 2016). Therefore, this randomized controlled trial was performed to explore the beneficial effects of SM on energy metabolism and compare the effect of SM with that of TMZ and control treatments in patients with HF. Using a standard protocol of SM injection and TMZ for HF (Chen et al., 2012; Fragasso et al., 2013; Xian et al., 2016), patients with HF were enrolled strictly according to the criteria, and the study protocol was strictly implemented to exclude all relevant factors that might interfere with the study results. We found that SM additionally improved cardiac function compared with the control treatment in HF patients following the drug doses. Moreover, markers of energy metabolism were improved after the SM treatment, and the improvement was even more remarkable than that following a standard treatment with TMZ.

We found that after treatment for 7 days, the cardiac function of the patients according to the NYHA functional classification was significantly improved from baseline and that the effects of SM were better than those of the TMZ or control treatment. In addition, we adopted a new indicator system established according to TCM syndromes to specifically quantify the clinical effects of TCMs (Mao et al., 2009; Wang et al., 2019b). This finding corresponded to the observed improvements in cardiac function. Furthermore, the BNP levels in the SM, TMZ, and control groups after treatment for 7 days were significantly lower than those at baseline in this study, while the effect of the SM treatment was the most obvious among the groups. Previous studies have shown that BNP, which is a specific indicator of left ventricular function, is proportional to ventricular dilatation and pressure overload (Martindale et al., 2016; Maisel et al., 2018). Overall, these findings show that compared with the control and TMZ treatments, SM is associated with more remarkable improvement in cardiac function in HF patients.

With decreased LVEF, myocardial energy consumption (MEE) increases, and the changes in the serum profiles of small-molecule metabolites are related to the pathophysiological mechanisms of elevated MEE in HF (Du et al., 2014). Our results demonstrate that the addition of SM to standard medical therapy in patients with HF (NYHA≥III) significantly decreased metabolic substrates in serum. After 7 days of treatment, the levels of FFA in the serum in the SM, TMZ, and control groups were significantly decreased, and the effect of SM was more obvious than that of TMZ and the control. We also found that the levels of serum LA in the SM, TMZ, and control groups were significantly decreased after 7 days of treatment, and no difference was observed among the groups. The PA content in the serum in the SM group was significantly lower than that at baseline after 7 days of treatment, but there were no changes in the TMZ and control groups. Previous research has shown that FFA oxidation is unchanged or slightly increased during the early stage of HF. However, in advanced- or end-stage HF, FFA utilization is decreased (Geltman et al., 1983; Osorio et al., 2002). With the aggravation of HF, glucose uptake and glycolysis are increased as a result of reduced oxidative metabolism (Nascimben et al., 2004). In addition, the serum level of FFA in patients with severe HF is significantly higher than the normal level, and serum FFA decreases after improvement in cardiac function in patients with end-stage HF (Salerno et al., 2015; Degoricija et al., 2019). Additionally, elevated LA is associated with poor tissue perfusion and/or oxygenation of blood in HF (Adamo et al., 2017), corresponding to the significantly decreased pumping function of the myocardium. Moreover, the serum levels of FFA and LA, which represent serological metabolism indexes, have been associated with mortality in HF patients (Gjesdal et al., 2018; Degoricija et al., 2019). In addition, it has been confirmed that as a metabolic substrate, PA is associated with enhanced myocardial free energy for ATP hydrolysis more than regular substrates in the myocardium, such as glucose, lactate and fatty acids, and the superiority of plasma PA seems to be more remarkable (Mallet et al., 2018). The increased oxidative metabolism of PA could partially compensate for energy deficiency in patients with HF (Gray et al., 2014). Therefore, the decrease in the LA level is accompanied by a decrease in the PA level, indicating that aerobic oxygenation in HF patients is enhanced, anaerobic glycolysis is weakened, and the blood circulation of the system is improved. Taken together, our results indicate that the levels of FFA, LA, and PA in the serum were reduced in parallel to the improvement in cardiac function in HF patients after the integrative treatment with standard medicines and SM, suggesting that the SM treatment optimized the energy substrate utilization of the circulatory system to improve the energy supply in HF patients, thus further improving heart function.

It has been observed that serum concentrations of BCAA in patients with HF are lower than those in healthy subjects (Tsuji et al., 2018). However, studies investigating end-stage HF found reduced plasma and tissue levels of amino acids (McKirnan et al., 2019). The level of amino acids increased after improvement in HF function and, therefore, could represent a compensatory mechanism and an alternative energy source that could fuel the TCA cycle *via* cataplerosis (Diakos et al., 2016). Our results show that the level of BCAA in the SM group was significantly increased after treatment for 7 days and that the effect of SM on BCAAs was superior to that of the TMZ and control treatments. Thus, as an auxiliary drug for the standard treatment of HF, SM can increase the BCAA content in circulation and provide an energy metabolism substrate for patients with HF to promote energy production. Furthermore, the effect of SM was more obvious than that of TMZ, which served as a positive control.

Our research results show that SM injection, which is a TCM used to tonify Qi, can improve myocardial energy metabolism in patients with HF, providing more evidence for the treatment of HF with compound Chinese medicines used to tonify Qi. This treatment used to correct the imbalance of energy metabolism may open up a new way to treat diseases related to energy metabolism disorders, such as HF and myocardial ischemia, with TCM.

### Study Limitations

This study has some limitations. First, although the purpose of this study was to compare the effects of SM as an auxiliary drug for HF treatment on metabolism in the body, the detected serological metabolic indexes were limited, and the correlations between the metabolic indexes and improved cardiac function and the relationships among the metabolic indexes were not directly observed. Therefore, these results should be confirmed by additional studies investigating changes in serological metabolism after SM treatment in HF. Second, the included patients were limited to those hospitalized at the Department of Cardiovascular Medicine at one hospital. Although the sample size of this study met the requirements of a randomized controlled trial, the relatively small sample may lead to a certain deviation in the results of this study. Thus, a study with a larger sample size involving multiple centers should be conducted to validate these findings. Finally, TMZ was used as a positive control to investigate the changes in serological metabolic indexes, and the treatment duration of TMZ in this study was shorter than that in previous studies. Additional comparisons between TMZ and SM after prolonging the course of treatment could be valuable.

## Conclusions

In summary, integrative treatment with SM in addition to standard medical treatment for HF was associated with improved cardiac function compared to standard medical treatment alone. The benefit of SM in HF may be related to improvement in energy metabolism, which seems to be more remarkable than that following treatment with TMZ. Furthermore, the results provide a new evaluation index for studies investigating TCM in the treatment of HF.

## Data Availability Statement

All datasets generated for this study are included in the article/supplementary material.

## Ethics Statement

The studies involving human participants were reviewed and approved by the Ethics Committee of Zhejiang Provincial People’s Hospital. The patients/participants provided their written informed consent to participate in this study. Written informed consent was obtained from the individual(s) for the publication of any potentially identifiable images or data included in this article.

## Author Contributions

Conceived and designed the experiments: L-HW. Performed the experiments: S-MW and L-FY. Analyzed the data: S-MW and L-FY. Wrote the manuscript: L-HW, S-MW, and L-FY. All authors read and approved the final manuscript.

## Funding

This study was supported by the National Natural Science Foundation of China (no. 81670447), the National Natural Science Foundation of Zhejiang Province (no. LY15H020006), the Zhejiang Province Key Subject of Medicine (Neurological Rehabilitation), and the Traditional Chinese Medicine Program of Zhejiang Province (no. 2017ZZ001). L-HW received funding from the Zhejiang Provincial Program for the Cultivation of High-Level Innovative Health Talents.

## Conflict of Interest

The authors declare that the research was conducted in the absence of any commercial or financial relationships that could be construed as a potential conflict of interest.
